# Do postage stamps versus pre-paid envelopes increase responses to patient mail surveys? A randomised controlled trial

**DOI:** 10.1186/1472-6963-8-113

**Published:** 2008-05-28

**Authors:** Katrina Lavelle, Chris Todd, Malcolm Campbell

**Affiliations:** 1 School of Nursing, Midwifery & Social Work, The University of Manchester, University Place, Oxford Road, Manchester, M13 9PL, UK

## Abstract

**Background:**

Studies largely from the market research field suggest that the inclusion of a stamped addressed envelope, rather than a pre-paid business reply, increases the response rate to mail surveys. The evidence that this is also the case regarding patient mail surveys is limited.

**Methods:**

The aim of this study is to investigate whether stamped addressed envelopes increase response rates to patient mail surveys compared to pre-paid business reply envelopes and compare the relative costs. A sample of 477 initial non-responders to a mail survey of patients attending breast clinics in Greater Manchester between 1/10/2002 – 31/7/2003 were entered into the trial: 239 were randomly allocated to receive a stamped envelope and 238 to receive a pre-paid envelope in with their reminder surveys. Overall cost and per item returned were calculated.

**Results:**

The response to the stamped envelope group was 31.8% (95% CI: 25.9% – 37.7%) compared to 26.9% (21.3% – 32.5%) for the pre-paid group. The difference (4.9% 95% CI: -3.3% – 13.1%) is not significant at α = 0.05 (χ^2 ^= 1.39; 2 tailed test, d.f. = 1; P = 0.239). The stamped envelopes were cheaper in terms of cost per returned item (£1.20) than the pre-paid envelopes (£1.67). However if the set up cost for the licence to use the pre-paid service is excluded, the cost of the stamped envelopes is more expensive than pre-paid returns (£1.20 versus £0.73).

**Conclusion:**

Compared with pre-paid business replies, stamped envelopes did not produce a statistically significant increase in response rate to this patient survey. However, the response gain of the stamped strategy (4.9%) is similar to that demonstrated in a Cochrane review (5.3%) of strategies to increase response to general mail surveys. Further studies and meta analyses of patient responses to mail surveys via stamped versus pre-paid envelopes are needed with sufficient power to detect response gains of this magnitude in a patient population.

## Background

Mail surveys are widely used both in the general population and specifically with patients to assess patient satisfaction and as part of clinical studies. This has advantages to surveying the patients at the hospital as patients are less likely to be influenced by the setting and may require less staff time [1]. Disadvantages include limitation to the complexity of the questions that can be included and potentially lower response rate [2]. Maximising responses is crucial to reducing response bias and to help ensure the validity and reliability of the study [3]. One suggested method of increasing response rates is the inclusion of stamped addressed envelopes rather than pre-paid envelopes to return the survey. The increased cost of stamps (as postage is paid for questionnaires that are not returned) may be offset by a greater response rate [4].

A Cochrane review identified 21 trials comparing response rates from postal surveys enclosing stamped envelopes versus pre-paid envelopes [5,6]. The odds of response to surveys using the stamped envelopes were found to be over a quarter higher compared to those using pre-paid envelopes (OR = 1.29, 95% CI: 1.18–1.42). However there was also significant heterogeneity between the trial results (P < 0.001). This may be explained by the variation in trial populations and limit the generalisability of the results of specific populations [6].

The majority of the studies included in the Cochrane review were published in the market research or educational research journals and those studies focusing on health research are mainly limited to surveys of health professionals or the general population. Only one trial out of 21 involved a patient survey; a small trial of 138 patients with acute stroke amongst whom a significant difference in returns via stamped versus pre-paid envelopes was not established (P = 0.786) [7]. Similarly, McColl et al's review identified six studies comparing stamps to pre-paid returns^8^. Three of these studies concerned health topics, all of which surveyed health professionals or the general population rather than patients [8].

Responses to patient surveys may differ from other surveys due to adverse treatment outcome [9], interest in or salience of the topic [6,8,10] as well as recent and ongoing involvement with the service surveying them [11]. The relevance of the results of more general reviews to patient surveys may therefore be limited, and the need for specific reviews of influences on responses to patient postal surveys has been highlighted [10]. One such systematic review has demonstrated that more intensive follow up strategies and shorter questionnaires improved response rates to patient surveys [10]. However, no randomised trials of patient surveys comparing stamped with pre-paid response envelopes were identified and the authors highlight the need for future research to test such methods of improving response rates in patient populations.

Balancing the respective costs of stamps versus pre-paid returns against potential increases in response is of particular interest within the context of the resource limitations of contemporary health services. A meta-analysis undertaken in 1987 presented evidence that stamped envelopes produce a greater and more cost effective response than pre-paid [12]. However, the studies included in this latter review are mainly market research and date from 1951 to 1983. Hence their generalisability to current health care settings is limited. McColl et al's 2001 review presented mixed results with one study concluding that stamps appear to be more cost effective and three others finding stamped envelopes cost more than business replies per questionnaires returned/sent [8]. Two additional studies of mail surveys of health professionals undertaken in the USA report that using pre-paid envelopes was more expensive than stamps per item returned [13,14]. However, caution in drawing conclusions from these studies is required as they include different items in the costs and postal payment systems differ between countries. Moreover, no identified research has compared costs of pre-paid versus stamped returns specifically for a patient survey.

The present study therefore investigates whether inclusion of a stamped addressed envelope (as opposed to a pre-paid reply envelope) increases responses to survey reminders in a UK-based patient population. Differences in costs per item returned are also examined.

## Methods

A randomised-controlled trial (RCT) was undertaken to determine whether postage stamps versus pre-paid envelopes increases patient response rates to a reminder mail survey. The RCT was nested within a wider study of health related quality of life amongst female patients, aged ≥ 65 years, attending breast clinics in 11 hospitals in Greater Manchester between 1/10/2002 – 31/7/2003. As the study reported here was nested within our main study, the sample size was constrained by the sample size needed to achieve the main studies objectives. Initially, 844 patients were sent a 6 page survey, measuring functional status (ELPHS ADL) [15], generic health status (SF-12) [16] and health-related quality of life (EORTC QLQ-C30) [17] and a covering letter. These were included with the letter informing them of their first appointment at the breast clinic and a first class stamped addressed envelope for return of questionnaires. As these letters were sent out from the 11 different hospitals in the study, an RCT of the first posting of the survey was not feasible as random allocation would have to have been undertaken at each of the 11 sites by appointments staff.

**Figure 1:**
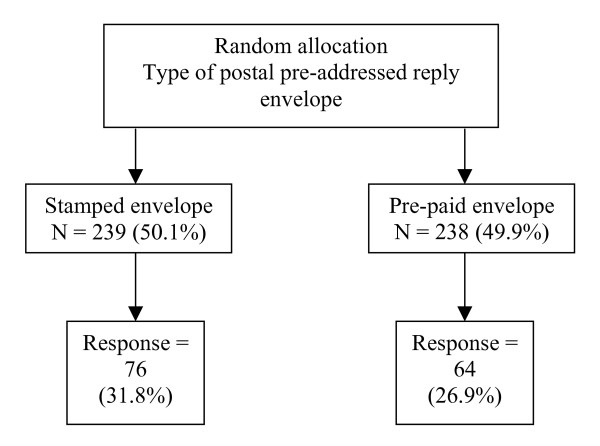
Response rates by allocation group.

Each survey was assigned a unique identifier. Hospital staff responsible for mailing surveys to patients kept a log book linking patients to survey identifiers. Surveys were returned to the University of Manchester where random allocation of non-responders could be undertaken. The 477 patients who had not returned the survey within 3 weeks were entered into the trial. The unique identifiers of these non-responders were randomly assigned, using SPSS 11.5 for Windows, to have either a first class stamped addressed reply envelope or a pre-paid addressed envelope included in their reminder letters. The random allocation for each non-responder's unique identifier was returned to the hospitals from where, each non-responder was sent a reminder covering letter along with another copy of the questionnaire and either a stamped or pre-paid response envelope. Of the 477 women, 239 were randomised to the group receiving the stamped envelope and 238 to the group receiving the pre-paid envelope (Figure 1). Although primarily determined by the number of non-responders, these sample sizes would have ensured that the study had 80% power to detect a difference of 12.5% (30% to 42.5%) at α = 0.05 using a two-sided uncorrected chi-squared test [18]. The cost per item returned and the overall cost of returns was calculated for both methods of response. The survey was approved by the North West Multi-Centre Research Ethics Committee (MREC/01/8/62).

## Results

The results of the RCT are illustrated in Figure 1. The overall response rate of initial non responders to this second mailing of the survey (with reminder) was 29.4% (140/477). The response rate was 31.8% for those in the stamped envelope group and 26.9% for the pre-paid group; a difference of 4.9% (95% CI: -3.3%–13.1%). Although the return rate for the stamped envelope group does appear to be higher, the 95% confidence interval of the difference includes zero and the return rate does not differ significantly, at a 5% two-sided level (χ^2 ^= 1.39; d.f. = 1; P = 0.239). There is, no evidence to reject the null hypothesis of no difference in return rates between those receiving the stamped and pre-paid envelopes. If the cost of setting up the (Royal Mail) freepost licence is included, the pre-paid method is more expensive at a cost of £1.67 per item returned versus £1.20 per item returned using stamped addressed envelopes and total cost of returns is £106.66 versus £90.82 respectively (Table 1). However, for some organisations the pre-paid licence will be considered a 'sunk' cost as one licence may be purchased for the whole organisation from which many surveys are undertaken. If the cost of the pre-paid licence is omitted the cost per item returned is less for the pre-paid returns (£0.73) compared to stamped returns (£1.20).

**Table 1:** Costs of stamped envelope returns vs. pre-paid envelope returns per item returned^1^

	**Stamped Envelope**			**Pre-paid envelope**		
	
	**Unit cost per item**	**Number of items**	**Total cost**	**Unit cost per item**	**Number of items**	**Total cost**
**Return first class post**	£0.27	239	£64.53	£0.27	64	£17.28
**Handling charge**	£0	0	£0	£0.05	64	£3.20
**Return envelope with printed address**	£0.11	239	£26.29	£0.11	238	£26.18
**Number of responses**		**76**			**64**	
**Pre-paid licence**	£0	0	£0	£60	1	£60
**Total cost including **pre-paid licence cost			**£90.82**			**£106.66**
**Cost per item returned including **pre-paid licence cost	£90.82/76 = **£1.20**			£106.66/64 = **£1.67**		
**Total cost excluding **pre-paid licence cost			**£90.82**			**£46.66**
**Cost per item returned excluding **pre-paid licence cost	£90.82/76 = **£1.20**			£46.66/64 = **£0.73**		

1. Costs contemporary to July 2002.

The marginal costs of each additional questionnaire returned were also calculated as follows. If the pre-paid licence cost is included, the overall cost is £15.84 cheaper for stamped versus the prepaid strategy. The stamped envelopes provided 12 extra returns compared to the pre-paid envelopes. The use of stamped envelopes therefore presents a saving of £1.32 per extra return (£15.84/12). However, if the cost of the pre-paid licence is excluded, the overall cost of stamped envelopes is considerably more expensive than pre-paid (£44.16). This presents a cost of £3.68 for every extra return (£44.16/12).

## Discussion

The results suggest that using stamped return envelopes rather than pre-paid replies does not significantly change the response rate to reminders for patient surveys. However, this study had 80% power to detect a difference of only 12.5% or more between the two groups; differences smaller than 12.5% would not be identified as significant in this study. Thus we need to treat our finding with some caution.

Edwards et al's Cochrane review found that stamped envelopes increase the odds of response by 1.29 (95% CI: 1.18–1.42) compared to pre-paid [6]. The response to the pre-paid envelopes in the present study (26.9%) would therefore be expected to increase to 32.2% according to the results of this review. Hence, our actual result, an increase in response to 31.8%, is consistent with the existing evidence.

Although this suggests that, as with other types of surveys, stamped envelopes increase responses to patient surveys compared to pre-paid envelops, this assumption needs to be tested in further RCTs of patient surveys with sufficient participants to detect a response gain of approximately 5% or more at 80% power. However, this would require large sample sizes. For example, the present study would have needed 1305 participants in each group to detect a difference of 5% (27%–32%).

An alternative approach would be undertake meta-analyses combining the results of smaller studies of patient returns to mail surveys, using similar methodology to the Cochrane review [6]. However, this review contained only one small study (n = 138) of a patient survey, therefore more trials of returns to patient surveys need to be undertaken before this sub analysis is viable. The lack of available evidence concerning strategies to improve returns to patient surveys has been highlighted in a recent review. Nakash et al combine 15 trials of strategies to improve responses to patient surveys and demonstrate that shorter questionnaires and more intensive follow up strategies can improve response [10]. However, no trials of stamped versus prepaid envelopes were identified and the authors stress the need to test other such strategies specifically within patient populations.

Our study suggests that the stamped envelope strategy is cheaper per returned item compared to pre-paid returns if set-up costs are included. Previous evidence suggests that the use of pre-paid envelopes is usually more expensive per item returned because of the additional return yielded by first class post [12] under these circumstances. However, if we omit the set up costs of the licensing fee on the basis that this is a fixed cost for an institution, the pre-paid envelope strategy is cheaper per item returned. This suggests that in an organisation undertaking a large number of surveys covered by one pre-prepaid licence, using a pre-paid system of survey returns may provide the cheaper option compared to using stamps.

Only women aged ≥65 years where eligible to receive the survey in this study. As response rates to mail surveys differ by both age and gender [3] the generalisability of the results to younger people and men may be limited. Differential response rates to return post methods may also exist between socio demographic groups. These could not be investigated in our study as we surveyed only older women. However, Shiono et al, found a significant difference for men (+5.9%) but not women [19]. Conversely, Harrison et al, found no difference in response rates to stamps versus pre-paid by age or gender [20]. Further studies of responses to these postal methods between various socio-demographic groups are needed.

This study investigated responses to a second mailing, primarily due to methodological constraints of randomising at first mailing. Although this may limit the generalisability of our findings to first and final response rates, as the Cochrane review found both first and final response rates are increased with stamps compared to pre-paid envelopes, this seems unlikely [6]. However, as this review selected studies randomised at first mailing conclusions specific to second or subsequent mailings can not be drawn. For example, the study undertaken by Shiono et al demonstrated a significantly higher returns for stamps versus pre-paid (P > 0.05) for second (39.1% versus 35.1%) but not first (52.8 versus 51%) mailings [19]. However, as randomisation was undertaken at first mailing the impact of confounders on responses to the second mailing may have biased this result. Studies randomising at second mailing such as the present study are needed to confirm if strategies to increase response rates demonstrated for first and final response rates in the Cochrane review are also true for responses to second mailings.

## Conclusion

Compared with pre-paid business replies, including stamped envelopes did not produce a statistically significant increase in response rate to this patient survey. However, because our study was nested within a substantive study of older women's health and sample size was constrained by the demands of that main study we only had sufficient power to detected differences of 10 – 12.5%. The actual response gain of the stamped strategy (4.9%), is similar to that demonstrated in the Cochrane review (5.3%) suggesting that use of stamped envelopes versus pre-paid may provide similar gains as those demonstrated for general mail surveys [6]. This needs to be confirmed in further studies or meta-analyses of patient responses to mail surveys via stamped versus pre-paid envelopes with sufficient power to detect response gains of this magnitude in patient populations.

## Competing interests

The authors declare that they have no competing interests.

## Authors' contributions

All authors read and approved the final manuscript. KL devised study design, undertook data collection and analysis and produced the manuscript. CT devised study design, advised on analysis and approved and contributed to drafts and final manuscript. MC advised on sample size and analysis and approved drafts and final manuscript.

## Pre-publication history

The pre-publication history for this paper can be accessed here:


